# Impact of corporate environmental uncertainty on environmental, social, and governance performance: The role of government, investors, and geopolitical risk

**DOI:** 10.1371/journal.pone.0309559

**Published:** 2024-08-27

**Authors:** Xiao Guo, Pengfei Cheng, Baekryul Choi

**Affiliations:** 1 Department of International Trade, Jeonbuk National University, Jeonju-si, Jeollabuk-do, Republic of Korea; 2 Department of Financial Management, Hubei University of Automotive Technology, Shiyan City, Hubei Province, China; Liaoning University, CHINA

## Abstract

Corporations face multifaceted environmental uncertainties (EU) in today’s dynamic global economic environment. Such uncertainties profoundly affect corporate operations and pose significant challenges to their environmental, social, and governance (ESG) performance. Therefore, using data from Chinese A-share listed corporations from 2009 to 2021, we empirically analyze the impact of the EU on ESG performance. The results demonstrate that the EU significantly negatively impacts ESG performance, indicating that corporations frequently find it difficult to implement and maintain high-standard ESG policies and practices effectively. Additionally, they reveal that the EU inhibits the improvement of ESG performance by increasing corporate financing constraints (KZ). Lastly, this study explores the role of government subsidies (GOV), investor attention (IA), and geopolitical risks (GPR) as moderating variables. We discover that GOV can mitigate the negative impact of the EU on KZ because they provide additional resources that help corporations maintain their ESG in uncertain environments. Furthermore, IA can reduce the adverse impact of KZ on ESG. Positive moderating effects result from ESG issues; the capital they provide effectively reduces corporate KZ, thus enabling corporations to maintain good ESG performance despite operating in highly uncertain environments. However, due to the increased asymmetry of information in economic markets caused by geopolitical tensions, GPR exacerbates the negative impact of the EU on ESG performance, thus leading to an increase in KZ. These findings offer new perspectives on understanding how these moderating effects affect corporate ESG strategies.

## 1 Introduction

Corporations’ environmental, social, and governance (ESG) performance has garnered significant attention from the public, investors, and regulatory bodies in recent years. ESG performance is an indicator of a corporation’s commitment to sustainable development and is considered a critical factor in its long-term prosperity. Therefore, some recent studies have begun to explore the impact of financial constraints, green innovation, and audit quality on ESG performance [1–3]. Simultaneously, driven by globalization and technological advances, the economic environment in which businesses operate has become increasingly complex and uncertain. Environmental uncertainty (EU), which encompasses market fluctuations, political changes, and technological innovations, has a significant impact on corporate strategic planning and operational decisions. To date, however, the impact of the EU on corporate ESG performance has not yet been sufficiently explored. The term “EU” denotes the volatility and unpredictability of the external milieu surrounding a corporation. The unpredictability of corporate value outcomes results from various factors, including fluctuations in market demand, technology, policy, suppliers, and other environmental factors [4]. The impact of the EU on corporations is multifaceted; it not only affects their financial performance but may also influence their strategic decisions and organizational structures [5]. Studies have also shown a close relationship between the EU and social sustainability, with external EU undermining sustainable investments by corporations [6].

Moreover, as a significant moderating effect, government subsidies (GOV), investor attention (IA), and geopolitical risk (GPR) may moderate the relationship between EU and ESG performance. They can provide corporations with additional resources [7], thereby helping them maintain or enhance ESG performance in uncertain environments. IA to ESG issues may facilitate corporations’ maintenance of good ESG performance even when facing uncertainty [8]. Conversely, GPR may exacerbate the negative impact of the EU [9], making it difficult for corporations to implement ESG strategies effectively. However, current research on the effects of the EU on ESG performance is limited, and existing studies often overlook the critical moderating effects of the EU on ESG.

This study explores how the EU affects corporate ESG performance and analyzes the moderating roles of GOV, IA, and GPR. Through this research, we hope to provide valuable insights for corporate managers, policymakers, and investors, which will help them make more effective decisions in uncertain environments and promote sustainable corporate development. Based on this study’s research objectives, we have selected China as our study subject. Being the world’s second-largest economy with a large and diverse market, China’s rapid economic development and complex market environment provide rich empirical data and a unique research background for studying the impact of the EU on corporate ESG performance. Moreover, China’s individual political and economic systems also make it distinctive in GOV, IA, and GPR aspects. In this context, studying corporate ESG performance can deepen our understanding of the EU’s impact on corporate ESG performance.

Therefore, we utilized data from Chinese A-share listed corporations from 2009 to 2021 to empirically analyze the impact of the EU on corporate ESG performance. The results indicate that the EU significantly inhibits ESG performance. Moreover, the EU significantly and negatively impacts all the sub-components of ESG. Secondly, the EU inhibits ESG performance by increasing financing constraints (KZ). Thirdly, this study explores the role of GOV, IA, and GPR as moderating variables. We test GOV as a moderating effect on the KZ model because GOV can provide additional resources that alleviate KZ faced by corporations, thereby enabling them to invest in and maintain their ESG performance even in uncertain environments. On the other hand, IA and GPR are tested as moderating effects on the ESG model because IA can influence investor behavior and reduce information asymmetry, while GPR can introduce additional risks and uncertainties affecting ESG strategies directly.

This study contributes to existing research in the following ways.

First, it expands on related theories. This study extends the theory on the impact of the EU on corporate ESG. Previous research has primarily focused on the effects of the EU on corporate financial performance, with limited exploration of ESG performance. Further, by analyzing how the EU directly affects corporate ESG performance and exploring its impact mechanisms, this study enriches the related theory and literature.

Second, we reveal the moderating effects. Further, this study examines the moderating roles of GOV, IA, and GPR in the relationship between EU and corporate ESG performance. We find that GOV and IA can mitigate the negative impact of the EU on corporate ESG performance, whereas GPR may exacerbate this negative impact. This finding provides a new perspective on how these external factors influence corporate ESG strategies.

Third, we consider practical implications. This study provides critical practical implications for corporate managers, policymakers, and investors. For corporate managers, it underscores the significance of valuing and optimizing ESG performance in uncertain economic environments. In turbulent market conditions, corporations should focus more on maintaining and enhancing their ESG performance, which not only aids in their long-term sustainable development but also enhances their overall value. For policymakers, this study demonstrates the significant role of GOV in promoting corporate ESG practices. Governments should provide subsidies and incentives, especially during periods of high uncertainty, to support corporate ESG practices and promote long-term sustainable development. This study highlights the crucial role of investors in fostering corporate ESG performance. By focusing on and investing in corporations with strong ESG performance, investors can stimulate improvements in ESG practices while generating robust long-term returns for their investment portfolios. Therefore, investors should continue to monitor and support corporations that excel in ESG, especially in the face of EU and KZ.

Lastly, our findings are significant for understanding the impact of GPR in the context of globalization. Particularly when developing their global ESG strategies, corporations need to better understand and address GPR. This will enable them to minimize the potential negative impacts of these risks on their financing and ESG performance.

## 2 Literature review

### 2.1 EU and ESG performance

Widely acknowledged in the field of corporate management, the EU is a crucial determinant of corporate strategy and performance. Factors such as market changes, technological advancements, political and legal environments, and economic fluctuations are all elements of the EU that have the capacity to introduce potential instability and unpredictability into corporate operations [10]. Based on transaction cost theory and signaling theory, it is hypothesized that corporations must internally adjust their resource allocation and governance structures while responding to external market and policy changes in the face of EU. This approach minimizes transaction costs and enhances ESG performance. By applying these theories, the impact of the EU on corporate ESG performance is analyzed.

From a business perspective, the relationship between a corporation and its environment is intricately interconnected [11]. In an orderly and stable business environment, corporations are better able to clearly discern the current market situation and make informed decisions. On the one hand, adapting to a complex market environment incurs additional costs. With limited total capital, corporations may be compelled to reduce their investment in ESG initiatives, which ultimately affects their ESG performance. On the other hand, in a stable business environment, corporations can invest in the ESG domain, thereby enhancing their ESG performance [12].

Moreover, studies have demonstrated that the EU may present difficulties for corporations in terms of signaling, thereby affecting their KZ and impacting ESG performance. High levels of EU may result in informational asymmetries between the corporation and the external environment. Changes in the external environment, characterized by repetitiveness, disorder, and unpredictability, exacerbate this information asymmetry [13]. For instance, external investors struggle to differentiate corporate information, which hinders their capacity to grasp transaction costs and make prudent investment decisions. Consequently, their investment interest is diminished.

In addition, the specific impacts of EU on ESG performance are as follows.

#### 2.1.1 Environmental performance

EU significantly impacts a company’s environmental performance [14]. Firstly, high EU increases the difficulty of investing in environmental technologies and establishing environmental management systems. Due to the uncertainty of future policies and market demand, corporations may delay or reduce investments in environmental technologies, thereby affecting their environmental performance [15]. Secondly, EU can lead to reduced efficiency in resource utilization and waste management, further impacting environmental performance. For example, market demand fluctuations may cause changes in production plans, leading to increased resource waste and pollution emissions [16].

#### 2.1.2 Social performance

EU also significantly affects a corporation’s social performance. Under high EU, corporations face greater operational pressure and may cut back on social responsibility investments [17]. For instance, corporations might reduce expenditures on employee training, community development, and charitable donations, thereby affecting their social performance. Furthermore, EU can impact labor relations and employee morale, subsequently influencing social performance [18].

#### 2.1.3 Governance performance

EU is also significantly related to corporate governance performance [19], mainly reflected in the increased complexity and uncertainty of managerial decision-making. High EU can lead to greater information asymmetry and decision-making risks for corporate management, potentially reducing governance efficiency [20]. Additionally, EU can affect a corporation’s internal controls and risk management mechanisms, making it difficult to effectively respond to external shocks, thereby impacting governance performance [21].

Based on this, we propose Hypothesis 1.

H1: As the corporate EU increases, its ESG performance will decline.

### 2.2 EU, KZ, and ESG performance

The EU comprises, among other things, market volatility, political instability, and technological change. Under high EU conditions, a corporation’s financing costs and difficulties may increase, subsequently affecting its ESG performance. From the perspective of financial theory, the EU elevates the firm’s risk, which could result in investors and lenders demanding higher risk premiums; consequently, this would raise the corporation’s KZ [22]. Moreover, heightened uncertainty can make predicting future cash flows more challenging, thereby increasing lenders’ concerns regarding a corporation’s financial stability and restricting its access to financing channels [23]. Such KZ may limit a corporation’s investment in ESG projects because they typically require substantial initial capital and have longer payback periods.

Moreover, according to the resource dependence theory, corporations rely on external resources to maintain their operations and competitive edge [24]. Under high EU, KZ could restrict a corporation’s ability to access necessary resources, thereby impacting the implementation and performance of its ESG projects. Inadequate funding may hinder a corporation’s investment in ESG initiatives [25]. Furthermore, institutional theory posits that the external environment constrains corporate behavior and decisions [26]. Constrained financing may necessitate that corporations adjust their strategies in response to this external pressure; this may result in reduced investment in particular ESG projects, thereby affecting their ESG performance. In summary, by increasing KZ, the EU indirectly impacts corporate ESG performance. Based on this, we propose Hypothesis 2.

H2: EU indirectly inhibits ESG performance by increasing corporate KZ.

Existing empirical research supports the negative impact of EU on corporate ESG performance. Chen et al. (2011) show that high EU significantly increases a corporation’s KZ, which in turn affects its environmental and social performance [27]. Luo and Bhattacharya (2009) find that EU suppresses corporate ESG investment, with companies often prioritizing cuts in ESG-related expenditures when facing uncertainty [28]. Additionally, Jia and Li (2020) indicate that under high EU, corporations find it more challenging to maintain effective governance structures, thereby affecting their overall ESG performance [29].

In conclusion, EU significantly impacts corporate ESG performance by increasing decision-making complexity and exacerbating KZ. Understanding the impact of EU on corporate ESG performance not only advances academic research but also provides theoretical support and practical guidance for corporations to develop effective ESG strategies under uncertain environments. Future research can further explore other possible mediating mechanisms and moderating factors to fully uncover the pathways through which EU affects corporate ESG performance.

However, despite the existing research on the impact of EU on corporate ESG performance, several gaps remain. Existing studies often focus on specific aspects such as environmental or social performance, lacking a comprehensive approach that considers all three dimensions of ESG simultaneously. Moreover, most research is based on data from developed countries, with limited exploration of emerging markets like China. Additionally, there is insufficient examination of other potential mediating mechanisms and moderating factors, such as GOV, IA, and GPR, in the relationship between EU and ESG performance. This study aims to address these gaps by providing a comprehensive analysis of EU’s impact on corporate ESG performance and exploring the moderating roles of GOV, IA, and GPR, using data from Chinese A-share listed corporations from 2009 to 2021. By doing so, this research not only enriches the existing literature but also offers valuable insights for corporate managers, policymakers, and investors.

### 2.3 Moderating effect of the GOV

The relationship between corporations and governments is a significant institutional factor for businesses [30]. As an economic policy tool, GOV significantly impacts corporate operations and strategic decision-making. GOV may play a crucial moderating role in the relationship between EU and corporate ESG performance. This phenomenon can be understood and analyzed from both the resource-based view and institutional theory perspectives.

From the resource-based perspective, GOV provides corporations with additional resources that can be used to mitigate the negative impacts of the EU and support investments and improvements in ESG [31]. For instance, government financial subsidies for environmental projects can help corporations cope with market and technological uncertainties while maintaining or improving their environmental management performance. Moreover, the GOV can support innovation and enhancements in social responsibility and internal governance structures, especially during economic turmoil.

Institutional theory emphasizes the role of GOV in influencing corporate behavior and norms [26]. Governments provide financial support and convey recognition and encouragement for certain ESG practices via subsidies. This facilitates the ability of corporations to better predict future markets and mitigates the impact of corporate EU on ESG [30]. This institutional framework can, to some extent, adjust and alleviate EUs [32], thereby encouraging corporations to perceive ESG performance as a pathway to long-term competitive advantage and market recognition.

To comprehensively understand the moderating role of GOV, it is essential to examine several key mechanisms through which these subsidies exert their influence.

Firstly, GOV can effectively alleviate KZ by providing necessary financial support, enabling firms to maintain and enhance their ESG performance even under high EU [33]. For example, subsidies can support the development of environmental technologies and social responsibility projects, thereby improving environmental and social performance [34].

Secondly, under high EU, the risks associated with ESG investments may lead firms to adopt a cautious approach. GOV reduce these risks, incentivizing firms to invest more in environmental protection and social responsibility [15]. By serving as a risk buffer for ESG investments, subsidies enhance a firm’s performance in these areas.

Finally, GOV play a significant role in enhancing corporate reputation. Firms receiving subsidies are often perceived as high-responsibility entities endorsed by the government, which not only boosts their market reputation but also strengthens their social responsibility image [18]. A good reputation can attract more investor interest, further improving ESG performance.

In summary, the moderating role of GOV is predicated on its capacity to provide corporations with additional resources to cope with the EU and institutionally promote improvements in ESG performance. Based on this, we propose Hypothesis 3.

H3: GOV mitigates the negative impact of EU on KZ.

### 2.4 Moderating effect of the IA

Research indicates that high-quality financial and social information disclosure can reduce a corporation’s financing costs by decreasing information asymmetry [35]. Particularly in today’s context, investors are increasingly inclined to invest in corporations that excel in ESG aspects. Partly, this tendency is influenced by their conviction that such corporations can yield not only financial returns but also generate positive social and environmental benefits, thereby effectively mitigating the risks associated with the EU [6]. From the perspective of transaction cost theory, corporations seeking funding incur various costs related to information search, negotiation, and contract enforcement. Information asymmetry problems may be amplified by the EU’s repetitive, disorderly, and unexpected nature [13]. Investing in such situations with the participation of investors who have an in-depth knowledge of ESG practices can significantly reduce the costs associated with seeking and securing corporate funding. This is because transactions with these investors may involve less information asymmetry and lower negotiation costs.

Furthermore, the stakeholder theory emphasizes that corporations should be accountable to shareholders and consider other stakeholders’ needs and expectations, including investors’ attention to ESG issues [36]. These concerns may serve as an external pressure, thus influencing corporate performance in ESG aspects. Likewise, according to resource dependence theory, investors are not only providers of capital but also important sources of information and legitimacy [24]. Therefore, to retain the support of investors who are overly concerned with ESG issues, businesses may be more inclined to improve their ESG performance.

The institutional theory provides an alternative perspective regarding how IA influences corporate behavior. In addition to market and resource constraints, social and cultural norms also exert an influence on corporate behavior, according to this theory [26]. In the growing trend in socially responsible investing and ESG investing, IA could become an institutional pressure that compels corporations to adopt higher ESG standards.

To comprehensively understand the moderating role of IA, several key mechanisms are examined. High-quality disclosure of financial and social information reduces information asymmetry between corporations and investors, lowering financing costs [35]. This effect is particularly pronounced under EU, where detailed ESG disclosures help investors make informed decisions and reduce the perceived risk of their investments. Investors increasingly prefer corporations that perform well in ESG aspects, believing these firms provide financial returns along with social and environmental benefits [37]. This preference underpins investor confidence, encouraging further investments even under high EU, thereby improving the corporation’s ability to secure funds for ESG projects. EU amplifies transaction costs due to the increased need for information search, negotiation, and contract enforcement [38]. Investors with in-depth ESG knowledge can mitigate these costs by reducing information asymmetry and negotiation complexities, facilitating smoother transactions and more secure funding for ESG initiatives. Resource dependence theory suggests that investors are crucial providers of capital, information, and legitimacy [24]. Corporations aiming to retain ESG-focused investors are likely to enhance their ESG performance, benefiting from the resources and legitimacy these investors provide. As ESG investing becomes more prevalent, IA creates institutional pressure, compelling corporations to adopt and maintain high ESG standards. This institutional pressure aligns corporate strategies with broader societal expectations, promoting sustainable business practices.

In summary, IA is a source of capital and resources, as well as a conveyor of norms and expectations. This attention can effectively alleviate the KZ corporation’s face and provide the necessary resources for implementing and maintaining practical ESG projects. Based on this, we propose Hypothesis 4.

H4: IA reduces the negative impact of KZ on ESG performance.

### 2.5 Moderating effect of GPR

GPR, such as international conflicts, political instability, and changes in transnational laws and policies, are critical factors that affect the operations of globalized businesses. In the relationship between EU and corporate ESG performance, GPR may play a significant moderating role. This role can be analyzed from the perspectives of political risk theory and global strategic management.

Political risk theory focuses on the impact of political changes on corporate decision-making and performance [39]. An increase in GPR may expose corporations to more significant uncertainties, particularly regarding cross-border operations and supply chain management. This risk may force corporations to reassess their ESG strategies, particularly in instances where political changes affect their operations and compliance in specific countries or regions.

From the global strategic management perspective, GPR requires corporations to consider more complex and diverse factors when formulating and implementing ESG strategies. In addition to adhering to the legal frameworks of various nations, global corporations must confront obstacles arising from political volatility and shifts in policy [40]. In such situations, corporations may need more flexible and varied ESG strategies to navigate the political environments of various countries and regions.

In high GPR environments, the degree of information asymmetry in economic markets increases [41]; therefore, this situation increases corporate uncertainty about future expectations. This prompts financial corporations to take measures [42] that may lead to higher financing costs for corporations [43] and also increase the difficulty of obtaining external financing. The costs of information search, negotiation, and risk management also rise, particularly when dealing with external capital sources. These increased costs and difficulties can hinder a corporation’s ability to invest in and maintain effective ESG initiatives.

Furthermore, empirical evidence supports the notion that GPR exacerbates the adverse impacts of KZ on corporate performance. It has been demonstrated that political risk significantly affects corporate financial performance on a global scale, highlighting the need for corporations to strategically manage these risks to sustain their ESG performance [44]. In such environments, the costs of information search, negotiation, and risk management also increase, especially when transacting with external capital sources. Based on this, we propose Hypothesis 5.

H5: GPR exacerbates the adverse impact of KZ on corporate ESG performance.

To address these gaps, we explore how the EU affects corporate ESG performance and analyze the moderating roles of GOV, IA, and GPR. We test GOV as a moderating effect on the KZ model. GOV provide crucial financial support that helps alleviate the KZ corporations face in uncertain environments. This support is critical because financial constraints are a direct barrier to investing in ESG initiatives, which often require significant capital. By reducing these constraints, GOV can indirectly support better ESG performance. Conversely, IA and GPR are tested as moderating effects on the ESG model itself. IA can influence corporate behaviour by reducing information asymmetry and encouraging investments that align with ESG principles. Investors who focus on ESG are likely to provide the necessary capital and support for corporations to maintain high ESG standards, even when facing KZ. GPR, on the other hand, add layers of complexity and uncertainty to the business environment. These risks can exacerbate the negative impact of the EU on ESG performance by increasing information asymmetry and the unpredictability of external conditions, thus directly affecting corporate ESG strategies.

## 3. Methodology specification and variable description

### 3.1 Model construction

#### 3.1.1 Baseline model

To empirically test the impact of the EU on ESG, we integrate the EU and ESG into a unified analytical framework based on the research hypothesis. The baseline model is as follows:

$${\text{ESG}}_{\text{it}}=\alpha_{0}+\alpha_{1}{\text{EU}}_{\text{it}}+\alpha_{2}\sum {\text{Controls}}_{\text{it}}+\varepsilon_{\text{it}}$$ (1)

where t and i represent the enterprise and year, respectively. ESG_it_ represents the ESG performance index; EU_it_ represents the environment uncertainty; Controls_it_ represents the set of control variables; and ε_it_ represents the error term.

### 3.1.2 Mediating effect model

Applying the mediation model allows us to delve deeper into the complex interactions among variables and gain a more nuanced understanding of the underlying mechanisms. In testing mediation effects, several academic studies have adopted the causal step regression analysis method proposed by Baron and Kenny (1986) [45]. In terms of analyzing mediation effects, this method is logically intuitive and straightforward, which makes it easy for researchers to articulate and readers to understand. To verify Hypothesis 2-the mediating role of KZ, we construct a corresponding mediation effect model based on the theoretical framework of Baron and Kenny (1986) [45].


$${\text{ESG}}_{\text{it}}={\text{a}}_{\text{0}}+{\text{a}}_{\text{1}}{\text{EU}}_{\text{it}}+{\text{a}}_{\text{2}}\sum {\text{Controls}}_{\text{it}}+\varepsilon_{\text{it}}$$ (2)

$${\text{KZ}}_{\text{it}}={\text{b}}_{\text{0}}+{\text{b}}_{\text{1}}{\text{EU}}_{\text{it}}+{\text{b}}_{\text{2}}\sum {\text{Controls}}_{\text{it}}+\varepsilon_{\text{it}}$$ (3)

$${\text{ESG}}_{\text{it}}={\text{c}}_{\text{0}}+{\text{c}}_{\text{1}}{\text{EU}}_{\text{it}}+{\text{c}}_{\text{2}}{\text{Kz}}_{\text{it}}+{\text{c}}_{\text{3}}\sum {\text{Controls}}_{\text{it}}+\varepsilon_{\text{it}}$$ (4)

In this model, KZ represents the mediating variable. If the coefficients b_1_ and c_2_ are significant, then KZ mediates the relationship between EU and ESG.

#### 3.1.3 Moderating effect model

To examine the moderating effects of GOV, IA, and GPR, we incorporate interaction terms into the baseline model based on theoretical analysis and research hypotheses. The specific model is as follows:

$${\text{KZ}}_{\text{it}}{\text{=d}}_{\text{0}}+{\text{d}}_{\text{1}}{\text{EU}}_{\text{it}}+{\text{d}}_{\text{2}}{\text{GOV}}_{\text{it}}+{\text{d}}_{\text{3}}{\text{EU}}_{\text{it}}\times {\text{GOV}}_{\text{it}}+{\text{d}}_{\text{4}}\sum {\text{Controls}}_{\text{it}}+\varepsilon_{\text{it}}$$ (5)

$${\text{ESG}}_{\text{it}}={\text{e}}_{\text{0}}+{\text{e}}_{\text{1}}{\text{EU}}_{\text{it}}+{\text{e}}_{\text{2}}{\text{KZ}}_{\text{it}}+{\text{e}}_{\text{3}}{\text{IA}}_{\text{it}}+{\text{e}}_{\text{4}}{\text{KZ}}_{\text{it}}\times {\text{IA}}_{\text{it}}+{\text{e}}_{\text{5}}\sum {\text{Controls}}_{\text{it}}+\varepsilon_{\text{it}}$$ (6)

$${\text{ESG}}_{\text{it}}={\text{f}}_{\text{0}}+{\text{f}}_{\text{1}}{\text{EU}}_{\text{it}}+{\text{f}}_{\text{2}}{\text{KZ}}_{\text{it}}+{\text{f}}_{\text{3}}{\text{GPR}}_{\text{t}}+{\text{f}}_{\text{4}}{\text{GPR}}_{\text{t}}\times {\text{KZ}}_{\text{it}}+{\text{f}}_{\text{5}}\sum {\text{Controls}}_{\text{it}}+\varepsilon_{\text{it}}$$ (7)

where GOV_it_ represents the government subsidy, IA_it_ represents the investor attention, and GPR_t_ represents the geopolitical risks.

### 3.2 Variable description and data source

This study focuses on sample data from Chinese A-share market-listed corporations between 2009 and 2021. To ensure the completeness and accuracy of the data, 29,976 valid observations were made. Basic information and financial data were primarily sourced from the China stock market & accounting research database, which is widely regarded as a crucial resource for researching the Chinese capital market.

#### 3.2.1 Dependent variable

The importance of ESG as a catalyst for sustainable development is widely recognized [46]. Thus, investors, the business community, and government corporations are placing a greater emphasis on ESG performance as an increasingly vital metric for measuring corporate performance in sustainable development [47]. Among various assessment tools, the Huazheng ESG rating is favored by scholars due to its extensive coverage and data completeness [48]. Based on this, we utilize the Huazheng ESG rating (https://www.chindices.com/) to evaluate corporate ESG performance.

#### 3.2.2 Core explanatory variable

The EU is the key variable of interest of this study. Given that a corporation’s operational status is significantly influenced by environmental changes, fluctuations in its performance can be an essential indicator for measuring the EU it faces. Based on the research by Ghosh and Olsen (2009) [49], we believe that the portion of abnormal growth in operating income, excluding the fixed growth component, can more accurately depict the EU’s situation. Based on this, our study adopts Model (8) to calculate the abnormal operating income of corporations over the past five years, employing this as a measure of the EU.


$$\text{Sale}=\varphi_{0}+\varphi_{1}\text{Year}+\varepsilon_{1}$$ (8)

Where Sale represents operating income, and Year denotes the year variable. If the data being examined is operating income from 5 years ago, the year variable is set to 1; if it is from 4 years ago, the year variable is set to 2, and so forth. As such, the residuals obtained from the linear equation qualify as abnormal operating income. We obtain a preliminary estimate of the EU by calculating the ratio of the corporation’s standard deviation of abnormal active income over the past five years to the average operating income for the same period. Additionally, by incorporating industry characteristics, we divide the result from the previous step by the industry median to calculate the environmental uncertainty value (EU2), which is tested for robustness as an alternative variable to the EU.

#### 3.2.3 Mediating variable

In measuring corporate KZ, scholars commonly use a multi-indicator approach to construct a KZ index. Utilizing the KZ Index to quantify the magnitude of KZ, this study employs the methodology of Lamont et al. (2001) to develop a proxy variable for KZ [50]. The constructing idea for the KZ index originates from the research of Kaplan and Zingales (1997) [51]. Building upon this, Lamont et al. (2001) employed five key variables [50], namely operating cash flow, Tobin’s Q, debt-to-assets ratio, dividend payout ratio, and cash holdings, to conduct an ordered logit regression analysis, thereby developing the KZ index. The level of the KZ index reflects the degree of KZ faced by listed corporations, with a higher value indicating greater difficulty in securing financing.

#### 3.2.4 Moderating variables

The moderating variables are as follows:

1. *GOV*. Numerous studies have indicated that government grants are key to a corporation’s growth phase. These subsidies share the risk when a corporation implements ESG strategies and provides a relatively relaxed testing ground, enabling it to experiment and adjust its strategies in a lower-risk environment [52]. Additionally, receiving GOV is frequently a direct result of a corporation establishing and strengthening political connections [53]. Based on these insights, this study selects the total GOV received by a corporation as the proxy variable for measuring GOV.
2. *IA*. Internet search volume directly indicates the degree to which a particular stock attracts IA. Research by Mbanga et al. (2019) demonstrates that the frequency of web searches for a stock can significantly reflect how much focus the stock is receiving among investors [54]. For the Chinese market, studies commonly use the Baidu index as the measurement tool because Baidu, which is China’s primary search engine, is extensively used by investors to search for stock market-related information [55]. Therefore, this study selects the Baidu Index using each stock’s name as a keyword as the proxy variable for measuring IA. Chen’s (2017) research also supports the rationality of this approach [56].
3. *GPR*. The GPR index is measured based on the research by Caldara and Iacoviello (2022) [57]. In constructing the GPR index, they analyzed the proportion of reports related to GPRs about total reports. These reports typically cover military threats, terrorism, domestic insurrections, and foreign aggressions [58]. Therefore, the GPR index is a comprehensive indicator encompassing all internal and external shocks, including wars and military tensions [59]. Because news reports are an essential channel for corporations and investors to obtain timely and reliable information, this index has been widely applied in recent studies [60, 61].

#### 3.2.5 Control variables

Consistent with existing research, our control variables include corporation size, the proportion of shares held by the largest shareholder, total operating revenue, tangible asset ratio, and leverage [62]. Table 1 presents the primary statistical analysis of each variable.

**Table 1:** Variables described.

Variables	Abbreviation	Obs.	Mean	Std. Dev.	Min	Max
Environment, society, and governance	ESG	29976	4.280	0.080	3.600	4.530
Environment	Environment	29976	4.080	0.130	3.380	4.560
Society	Society	29976	4.290	0.160	1.590	4.610
Governance	Governance	29976	4.360	0.120	2.840	4.580
Environmental uncertainty	EU	29976	-2.260	0.830	-6.890	0.470
Financing constraints (KZ index)	KZ	29976	0.510	0.970	-7.970	2.610
Government subsidies	GOV	29976	17.14	1.680	6.400	24.13
Investor attention index	IA	29976	12.65	0.740	6.230	17.29
GPR index (The logarithm of GPR index)	GPR	29976	-0.540	0.350	-0.970	-0.090
Corporation Size	SIZE	29976	22.16	1.330	15.04	28.64
Shareholding ratio of the largest shareholder	SR	29976	3.420	0.480	-1.240	4.500
Total operating income	TOI	29976	21.51	1.500	11.86	28.72
Tangible asset ratio	TAR	29976	-0.090	0.130	-2.210	0.000
Leverage (Total liabilities / total assets)	LEV	29976	-0.980	0.630	-4.950	4.060

As shown in Table 1, the average, maximum, and minimum values of ESG are 4.280, 4.530, and 3.600, respectively. This indicates that the ESG performance of A-share listed corporations in China is balanced, highlighting the importance of ESG in corporations. The EU’s average, maximum, and minimum values are -2.260, 0.470, and -6.890, respectively, indicating significant differences in the EU faced by listed corporations, which inevitably affect various corporate activities. Furthermore, the maximum and minimum values of KZ are 2.610 and -7.970, respectively, indicating considerable differences in KZ among the sample corporations. Additionally, information on other variables is consistent with existing research and aligns with the actual situation of the corporations.

## 4 Findings and discussions

### 4.1 Baseline results

Table 2 reports the regression results of the baseline model. In the first column of Table 2, only EU is used as the explanatory variable, whereas all control variables are included in the second column. Additionally, columns 3–5 of Table 2 report EU’s regression results on the ESG sub-components. In the results of columns 1–5, the coefficients of the EU are all significantly negative. In addition, the maximum value of the variance inflation factor (VIF) test is 5.25, which is less than 10. This indicates that there is no significant multicollinearity problem in our model. The coefficient of EU in column 2 is -0.007 and is significant at the 1% level. This indicates that for every 1% increase in the EU, corporate ESG performance decreases by 0.7%. Consistent with our expectations, the EU inhibits the growth of ESG performance (confirming Hypothesis 1). Moreover, the EU significantly negatively impacts ESG. This could be because, first, an increase in the EU results in increased information search and coordination costs for corporations, i.e., increased transaction costs. However, due to limited corporate resources, these additional costs may make it difficult for corporations to maintain high levels of ESG investment and practice, thus reducing ESG performance [6]. Second, an increase in the EU makes investors more cautious [63], which results in a reduction of corporate financial support. Consequently, as KZ increases, financing becomes more complex [51], and thus corporate ESG performance declines. Therefore, when the EU is high, corporations may reduce ESG activities, decreasing corporate ESG performance.

**Table 2:** Baseline results.

	(1)	(2)	(3)	(4)	(5)
	ESG	ESG	Environment	Society	Governance
EU	-0.007***	-0.007***	-0.003**	-0.005***	-0.010***
	(0.001)	(0.001)	(0.001)	(0.001)	(0.001)
SIZE		0.013***	0.013***	0.013***	0.014***
		(0.002)	(0.003)	(0.004)	(0.003)
SR		0.016***	-0.002	-0.004	0.037***
		(0.003)	(0.004)	(0.005)	(0.004)
TOI		0.007***	0.002	0.015***	0.005*
		(0.002)	(0.002)	(0.003)	(0.003)
TAR		0.008	0.009	-0.022*	0.022**
		(0.007)	(0.010)	(0.012)	(0.011)
LEV		-0.020***	0.006**	-0.001	-0.044***
		(0.002)	(0.002)	(0.003)	(0.003)
Constant	4.265***	3.754***	3.765***	3.674***	3.767***
	(0.002)	(0.034)	(0.047)	(0.057)	(0.054)
Firm fixed	YES	YES	YES	YES	YES
Year fixed	YES	YES	YES	YES	YES
Industry fixed	YES	YES	YES	YES	YES
R2	0.559	0.579	0.696	0.557	0.473
N	29976	29976	29976	29976	29976

Note: Standard errors in parentheses; *, **, *** represent level of significance at 10%, 5%, and 1% respectively.

Additionally, the control variables also provide insightful results. For instance, firm size (SIZE) positively affects ESG performance, indicating that larger firms are better able to manage ESG issues. The shareholding ratio (SR) has a mixed impact on the sub-components of ESG, suggesting that ownership structure plays a complex role. Total operating income (TOI) positively influences ESG, indicating that higher revenues enable better ESG practices. Leverage (LEV) generally has a negative impact on ESG, which may reflect the financial pressure leveraged firms face.

### 4.2 Mechanism and moderating effects analysis

Table 3 reports the mediation effect regression results. Firstly, columns 1–2 of Table 3 demonstrate that an increase in EU leads to a rise in KZ and a decrease in ESG. The EU can indirectly adversely affect ESG performance by increasing the KZ of the enterprise (confirming hypothesis 2). This may be due to the challenges in signaling for corporations when the EU is high, which impact their KZ and thus affects ESG performance. When corporations face high EU, the resulting information asymmetry makes it difficult for investors to distinguish corporation information [13], hindering their ability to grasp transaction costs and thereby reducing their investment interest. Essentially, a rise in EU increases KZ faced by corporations, subsequently affecting their ESG performance.

**Table 3:** Mechanism and moderating effects results.

	(1)	(2)	(3)	(4)	(5)
	KZ	ESG	KZ	ESG	ESG
EU	0.031**	-0.006***	0.224**	-0.006***	-0.005***
	(0.010)	(0.001)	(0.092)	(0.001)	(0.001)
KZ		-0.002***		-0.023**	-0.005***
		(0.001)		(0.010)	(0.001)
GOV			-0.011		
			(0.013)		
EU*GOV			-0.011**		
			(0.005)		
IA				-0.011***	
				(0.002)	
KZ*IA				0.002**	
				(0.001)	
GPR					-0.002
					(0.002)
KZ*GPR					-0.008***
					(0.002)
SIZE	-0.044*	0.016***	-0.057**	0.018***	0.006**
	(0.024)	(0.002)	(0.025)	(0.003)	(0.002)
SR	-0.162***	0.013***	-0.187***	0.012***	0.020***
	(0.032)	(0.003)	(0.032)	(0.003)	(0.003)
TOI	-0.142***	0.005**	-0.146***	0.006**	0.007***
	(0.019)	(0.002)	(0.021)	(0.002)	(0.002)
TAR	0.121	0.012	0.115	0.008	0.010
	(0.090)	(0.009)	(0.092)	(0.090)	(0.008)
LEV	1.153***	-0.023***	1.185***	-0.024***	-0.023***
	(0.038)	(0.003)	(0.038)	(0.003)	(0.003)
Constant	6.101***	3.742***	6.766***	3.822***	3.892***
	(0.382)	(0.043)	(0.451)	(0.053)	(0.037)
Firm fixed	YES	YES	YES	YES	YES
Year fixed	YES	YES	YES	YES	NO
Industry fixed	YES	YES	YES	YES	YES
R2	0.517	0.589	0.519	0.609	0.580
N	29976	29976	29976	29976	29976

Note: Standard errors in parentheses; *, **, *** represent level of significance at 10%, 5%, and 1% respectively.

Column 3 of Table 3 shows that the interaction term coefficients of GOV and EU are significantly negative. This means that GOV can mitigate the impact of the EU on corporate KZ (confirming Hypothesis 3). This is because relevant corporations’ institutional norms and policy support encourage the sharing of information and resources between enterprises [1]. Moreover, corporations’ institutional norms are often intricately linked with government policy support. In a competitive market environment, such corporations frequently encounter less competitive pressure, which alleviates the negative impact of uncertainty from competitors on the corporation. Additionally, the network of cooperative relationships established between the government and corporations helps reduce the costs of market transactions [64]. Therefore, government intervention reduces transaction costs and weakens the uncertain factors in the operating environment, thereby increasing corporate investment in ESG projects and improving ESG performance.

Thirdly, column 4 of Table 3 indicates that the coefficient of the interaction term between IA and KZ is significantly positive. This means that an increase in IA helps to reduce the negative impact of KZ on ESG; thus, it confirms Hypothesis 4. This may be because more investors are paying attention to corporations, thus reducing the risk compensation premium demanded by corporations and reducing the cost of debt and equity financing [65]. In other words, decreasing financing difficulties promotes progress in ESG aspects and enhances ESG performance.

Fourthly, column 5 of Table 3 shows that the interaction term coefficients of GPR and KZ are significantly negative. This implies that an increase in GPR exacerbates the adverse impact of KZ on ESG; thus, it confirms Hypothesis 5. The likely reason is that GPR, as a critical uncertainty factor, worsens the business and investment environment, reducing actual economic output, and causes capital market fluctuations [66]. In a business environment facing increased GPR, a corporations ability to obtain funding may vary. An increase in GPR intensifies the information asymmetry between corporations and external investors. This makes it difficult for fund providers to timely and accurately assess a corporation’s actual operational status, thereby disrupting investors’ capital allocation decisions [67]. Given the limited funding available to corporations, the funds allocated for ESG projects decrease, resulting in a decline in ESG performance.

The control variables such as SIZE, SR, and TOI maintain their significance and direction similar to Table 2, reinforcing the robustness of our findings across different models. These control variables highlight the importance of firm-specific factors in influencing ESG performance and financial constraints.

### 4.3 Robustness test

To ensure the robustness of our regression results, we implemented the following robustness testing methods:

1. Substituting EU: For robustness analysis, this study replaced the original EU variable with the industry-adjusted environmental uncertainty index (EU2). The first column of Table 4 presents the related results.
2. Substituting ESG performance. For further robustness testing, we utilized ESG data provided by Bloomberg from 2011 to 2021 to reduce bias in ESG evaluations by different corporations. The second column of Table 4 shows the results of this test.
3. Adding macro control variables. Following the study by Feng et al. (2023) [68], we introduced additional macro control variables to reduce potential biases due to omitted variables. The third column of Table 4 lists the results of this part of the robustness tests.
4. Endogeneity analysis. We used the two-stage least squares (2SLS) method to address potential endogeneity issues. As an instrumental variable for the 2SLS analysis, we employed the lagged one-period value of the EU. The fourth column of Table 4 presents the results.

**Table 4:** Robustness test results.

	(1)	(2)	(3)	(4)
	ESG	ESG	ESG	ESG
EU	-0.007***	-0.019***	-0.007***	-0.010***
	(0.001)	(0.005)	(0.0007)	(0.001)
SIZE	0.013***	0.298***	0.011***	0.015***
	(0.002)	(0.018)	(0.002)	(0.001)
SR	0.016***	-0.177***	0.019***	0.017***
	(0.003)	(0.024)	(0.003)	(0.002)
TOI	0.007***	-0.018	0.007***	0.009***
	(0.002)	(0.017)	(0.002)	(0.001)
TAR	0.009	0.112*	0.005	0.002
	(0.007)	(0.066)	(0.007)	(0.005)
LEV	-0.020***	-0.103***	-0.020***	-0.022***
	(0.002)	(0.019)	(0.002)	(0.001)
GDP			-0.055**	0.092***
			(0.018)	(0.017)
OPEN			0.134***	-0.007***
			(0.013)	(0.002)
Under-identification test				5026
				[0.000]
Weak identification test				6409
Constant	3.772***	-2.718***	3.811***	3.865***
	(0.034)	(0.340)	(0.037)	(0.017)
Firm fixed	YES	YES	YES	YES
Year fixed	YES	YES	NO	YES
Industry fixed	YES	YES	YES	NO
R2	0.579	0.649	0.574	0.107
N	29976	10113	29976	26579

Note: Standard errors in parentheses; P-values in square brackets; *, **, *** represent level of significance at 10%, 5%, and 1% respectively.

The results in columns 1–3 of Table 4 show that, in various robustness tests, the EU is significantly negatively correlated with ESG, which is consistent with the baseline model results. Moreover, in column 4, the under-identification test and weak identification test statistics indicate the validity of our 2SLS regression results. Moreover, the EU coefficient remains significantly negative, indicating that our baseline model does not have serious endogeneity issues and that the results are robust.

## 5 Conclusions

This study investigates the impact of corporate EU on ESG performance. In this process, it specifically analyzes the moderating roles of GOV, IA, and GPR. Through theoretical and empirical research, we have revealed how the EU affects corporate ESG performance by influencing a corporation’s KZ and how external factors moderate this impact. Thus, utilizing data from Chinese A-share listed corporations from 2009 to 2021, we have empirically analyzed the impact of the EU on ESG performance. The results demonstrate that the EU significantly negatively affects ESG performance, which indicates that corporations frequently struggle to effectively implement and maintain high-standard ESG policies and practices in uncertain environments. Secondly, our findings demonstrate that the EU inhibits improving ESG performance by increasing corporations’ KZ. Finally, this study explores the role of GOV, IA, and GPR as moderating variables. The research found that the GOV can mitigate the negative impact of the EU on KZ. This is because the government provides additional resources to help corporations maintain their ESG in uncertain environments.

Moreover, IA can reduce the adverse impact of KZ on ESG. The investors’ focus on ESG issues plays a positive moderating role, with the capital they provide effectively reducing corporate KZ. Thus, this enables corporations to maintain good ESG performance even in highly uncertain environments. However, GPR exacerbates the negative impact of the EU on ESG performance, which may be due to increased information asymmetry in economic markets caused by geopolitical tensions, leading to an increase in KZ. The presence of GPR intensifies the negative impact of the EU on ESG performance. Political instability and international conflicts may lead to resource redistribution, thereby affecting corporate ESG strategies and practices. Nevertheless, this also provides corporations with opportunities to enhance ESG performance, especially in instances where political changes drive higher ESG standards.

This study’s findings have significant implications for corporate managers, policymakers, and investors. For corporate managers, it is crucial to understand the impact of the EU on ESG performance and how to mitigate these effects through various strategies. Policymakers should consider designing and implementing effective subsidy policies to support corporate ESG practices. For investors, they should recognize their potential influence in driving corporations to enhance ESG performance.

This study has some limitations. Firstly, it relies primarily on data from Chinese A-share listed corporations, which may limit the general applicability of the research conclusions. When facing EU, corporations in different markets or countries might exhibit different response patterns. Secondly, although we have used several moderating variables, such as GOV, IA, and GPR, these may not comprehensively cover all factors influencing ESG performance. Lastly, the study’s timeframe is limited to 2009–2021; thus, it does not consider longer-term dynamic changes. Future research should overcome these limitations by expanding the sample scope, introducing new variables, and employing different methodologies to further deepen the understanding of this field.

## Supporting information

S1 Data (DTA)
